# Examining child schooling/care location and child temperament as predictors of restaurant-related behaviors during the COVID-19 pandemic: findings from a nationally representative survey

**DOI:** 10.3389/fnut.2024.1281686

**Published:** 2024-08-07

**Authors:** Juliana Goldsmith, Mackenzie J. Ferrante, Sara Tauriello, Leonard H. Epstein, Lucia A. Leone, Stephanie Anzman-Frasca

**Affiliations:** ^1^Jacobs School of Medicine and Biomedical Sciences, University at Buffalo, Buffalo, NY, United States; ^2^School of Environmental and Biological Sciences, Rutgers, The State University of New Jersey, New Brunswick, NJ, United States; ^3^Center for Ingestive Behavior Research, University at Buffalo, Buffalo, NY, United States; ^4^School of Public Health and Health Professions, University at Buffalo, Buffalo, NY, United States

**Keywords:** temperament, restaurants, children, COVID-19, childcare

## Abstract

**Purpose:**

Emerging research highlights impacts of the COVID-19 pandemic on U.S. families, including changes in eating behavior and increased child body mass index. Aims of the present study were to examine whether child temperament and at-home vs. out-of-home childcare/school predicted families’ restaurant-related behaviors during the pandemic. Examining energy balance-related behaviors, like restaurant patronage, during the pandemic can help better understand lasting impacts on child health behaviors and health outcomes.

**Methods:**

An online survey was administered to U.S. parents with a 4-to-8-year-old child in October 2020 (n = 1,000). Linear and logistic regression examined whether child temperament and at home vs. out-of-home childcare/school predicted: (1) the frequency the child consumed restaurant meals (take-out, delivery, dine-in), (2) who chose the child’s restaurant meal, and (3) parent-reported reasons for the child’s meal choice. Income, education, employment, race/ethnicity, and regional COVID-19 restrictions were tested as covariates.

**Results:**

Parents with children higher on negative affectivity reported more frequent restaurant use in-person (*p* < 0.05) and via delivery (*p* < 0.05) compared to parents of children lower on negativity. Child negativity was also linked with parent-reported reasons for children’s restaurant meal choices. Parents of children receiving at-home childcare/schooling used delivery services less frequently than those receiving out-of-home care or schooling (*p* < 0.01).

**Conclusion:**

These findings suggest that individual and family factors may impact restaurant use and the meal selection process for children using restaurants during and beyond the COVID-19 era. Continued examination of individual differences in the impacts of the COVID-19 pandemic can facilitate intervention and policy approaches that fit with different families’ needs.

## 1 Introduction

In March 2020, restrictive measures were put in place in the United States (U.S.) to slow the spread of the SARS-CoV-2 virus. As businesses and schools closed, families’ lives were disrupted with many parents providing child-care and schooling at home, often in combination with remote work (1, 2). Emerging research highlights potential lasting impacts of these well-intended but drastic changes to family routines, including on children’s diets and obesity risk (3). Similarly, a recent review identified continued impacts of the COVID-19 pandemic on various aspects of children’s health and well-being and also acknowledged that impacts may vary by individual factors (4).

Before the pandemic, children in the U.S. were commonly consuming energy dense and nutrient poor foods (5), and restaurants were a normative eating context for families. While there have been some efforts to improve the nutritional quality of foods offered to children at restaurants, restaurant meals still tend to be higher in calories and lower in nutritional quality than meals prepared at home (6), highlighting child restaurant use and restaurant meal selection as health-related behaviors of interest. Pre-pandemic, over one-third of children in the U.S. typically consumed fast food on a given day and, on average, about one-third of their daily energy intake came from fast food and quick and full-service restaurants (7, 8). However, there is emerging evidence that the pandemic may have impacted where and how Americans obtain their food, with increased home cooking and reduced restaurant use (9–12) overall, and some initial evidence of variability by sociodemographics. Our recent study found that about half of children were eating restaurant food at least 2–3 times per month in Fall 2020, with lower use and lower perceived safety of restaurants among some sociodemographic groups (9). For example, parents with lower education levels and lower income reported less take-out and delivery (9). There is a need for additional research to better understand inter-individual variability in children’s restaurant use and food selection during COVID-19. Whether children are spending their days at home vs. out-of-home childcare or school, as well as child temperament, have been linked with eating behavior generally (13–15) and may predict restaurant-related behaviors during the pandemic; however this has not been studied yet.

In 2019, only 3% of children were homeschooled while the remaining 97% of the 50 million children enrolled in primary or secondary education in the U.S. attended school in-person (16). Therefore, children receiving all of their schooling/care at home during the COVID-19 pandemic can be considered to be indicative of the aforementioned major shifts to children’s contexts and routines during this time (9). Brazendale et al. (3) highlight ways in which these drastic shifts in structure and routines are likely to impact “obesogenic” behaviors, including dietary intake. With children spending more time at home, the amount and types of food in the home become even more influential for children’s overall dietary intake (17). During the pandemic, families were buying a greater quantity of food for their home, including more high-calorie snack foods, desserts, and sweets, as well as nonperishable processed foods (15). Several studies have compared child behaviors during the pandemic to behaviors in summer months when changes in child eating, physical activity and routines lead to weight gain (18). During the pandemic, it was found that parents were more concerned about their child’s weight, and food insecure families were more impacted by school closures as school meals provide children with daily meals and necessary nutrients (2, 15). Children spending more time at home could impact food security, food acquisition, and food preparation.

Families’ restaurant-related behaviors may also differ by child temperament, or differences in reactivity and self-regulation (19). Such differences in children’s behavioral styles may evoke different types of feeding behaviors from caregivers and affect child eating behaviors and weight trajectories (14, 20). A recent study found that child negative affectivity was associated with less parent responsiveness which in turn resulted in poorer mealtime structure and quality (21). Additional research has shown a relationship between child temperament and weight-related outcomes specifically negative affectivity where higher levels of negative affectivity in young children is predictive of binge eating, emotional eating, stress-induced eating and obesity later in life (14, 22). Negative affectivity may also predict child eating styles such as “picky eating” because children with greater reactivity may limit their exposure to new foods and display more negative reactions to trying new tastes (22). This is important because selective eating places children at risk for both poor nutrition and poor eating habits. Additionally, parents of children higher in temperamental negativity were more likely to use instrumental and emotional feeding methods (22). Instrumental feeding is defined as rewarding a child with food for desired behaviors, and emotional feeding is the use food to soothe or distract a child even if they are not hungry. Both instrumental and emotional feeding have been associated with a higher body mass index as well as unhealthy food choices (22). Since parent feeding and child eating behaviors may differ by child temperament (22), it is possible that restaurant-related behaviors may also differ by child temperament during COVID-19.

Taken together, prior research suggests consuming food from restaurants is linked with children’s diet quality and overall energy intake, and that variability exists in the extent to which families consumed food from restaurants during COVID-19. As such, the primary goal of this research was to understand the extent to which school/care context and child temperament predicted variability in restaurant-related behaviors during COVID-19. Outcomes of interest were: (1) restaurant frequency (how often the child had food from restaurants via take-out, delivery or dining in), (2) factors driving children’s restaurant meal choices, and (3) whether or not the parent played a role in choosing the child’s meal. It was hypothesized that parents with children higher on temperamental negativity and children attending school or receiving care at home would use take-out and delivery from restaurants more frequently during COVID-19. It was predicted that the use of restaurants in-person would generally be low and would not differ based on the aforementioned factors. There were no *a priori* hypotheses linking temperament and child-care/schooling with the other restaurant-related outcomes of interest.

## 2 Methods

Study procedures are described in more detail and published elsewhere (9). This was an observational, cross-sectional survey study. Invitations to participate in this one-time online survey were sent to a stratified random sample using Harris Poll Online opt-in panel which includes millions of respondents that have agreed to participate in survey research.

### 2.1 Participants

Participants that identified as U.S. residents over the age of 18 years old with at least one 4-to-8-year-old child (*n* = 1,000) were invited to participate. To be eligible for the survey, participants needed to be English-speaking, be a parent/caregiver 18 years of age or older with at least one 4-to-8-year-old child, and have internet access. Demographic and contextual variables are reported in Table 1.

**Table 1:** Participant characteristics for study sample, weighted to be representative of parents with 4-8-year-old child(ren) in the U.S. (*n* = 1000).

	% (*n*)	Mean (SEM)		% (*n*)	Mean (SEM)
Participant Demographics					
**Gender**			**Race and ethnicity**		
Female	55 (548)		White	69 (693)	
Male	45 (446)		Black or African American	12 (124)	
Transgender	1 (9)		Asian	11 (105)	
Other	0 (4)		Other	8 (77)	
**Marital Status**			Hispanic	22 (216)	
Now married/Living with partner	83 (827)		**Highest level of education completed**		
Single/never married	9 (93)		≤ High School/GED	20 (192)	
Divorced/separated/widowed	8 (81)		Some college/Tech/associates	37 (374)	
**Age (y)**		38.8 (9.5)	Bachelor’s degree	15 (154)	
18–24	2 (22)		≥ Graduate degree	26 (262)	
25–34	31 (309)		**Government benefits received at any point in 2020**		
35–44	45 (452)		SNAP^a^	42 (413)	
45–54	14 (138)		WIC^b^	30 (300)	
55 +	8 (79)		Medicaid	46 (461)	
**BMI**			Disability	21 (211)	
≥ 25.0	33 (334)		TANF^c^	25 (248)	
≥ 30.0	17 (166)		**Number of children in the household**		2.4 (1.2)
**Current employment status**			1	18 (184)	
Employed full time	63 (632)		2	45 (452)	
Employed part time	6 (62)		3	24 (238)	
Self-employed	6 (66)		4+	12 (126)	
Not employed	7 (65)				
Homemaker/Stay-at-home	14 (141)		**4-8-year-old child with most recent birthday**		
**Household income (per year)**			Age – years		6.2 (1.4)
< $24, 999	10 (100)		Gender - % Male	55 (353)	
$25,000 – $34,999	7 (67)		**4-8-year-old-child eligible for free or reduced-price meals in school (*n* = 593)**		
$35,000 – $49,999	11 (110)				
$50,000 – $74,999	16 (165)		Yes	59 (353)	
$75,000 - $99,999	15 (149)		No	31 (186)	
>$100,000	41 (410)		Do not know	9 (54)	

Participant reported COVID-19 related restrictions in place in the town or city where the parent resides.			
Restrictions in town		Restaurant restrictions	
Most businesses are closed	18 (179)	Can offer take-out or delivery	29 (289)
Some businesses are closed	44 (444)	In-person allowed:	
Very few businesses are closed	38 (378)	Outdoors only	15 (150)
**Masks mandated**		Both outdoors and indoors: reduced capacity	50 (500)
Yes	90 (897)	Both outdoors and indoors: full capacity	6 (61)

Child school/care location			
Child school/care	February 2020%(n)	Last week %(n)	N/A %(n)
In-person Elementary School	59 (525)	21 (212)	29 (288)
Virtual Elementary School	30 (296)	47 (467)	29 (293)
Homeschooled	28 (279)	31 (310)	47 (471)
Child-care outside the home/ elementary school	26 (262)	19 (187)	60 (596)
Regular care in the home from someone other than parent/guardian	28 (281)	24 (236)	56 (561)

a SNAP—Supplemental Nutrition Assistance Program. b WIC—Special Supplemental Nutrition Program for Women, Infants, and Children cTANF—Temporary Assistance for Needy Families.

### 2.2 Procedures

Survey questions were fielded in October 2020, as part of a larger study designed to examine how families with 4-to-8-year-old children use restaurants (e.g., frequency of take-out/delivery, meal ordering behaviors) during the COVID-19 pandemic. The survey was created by researchers at the University at Buffalo. Researchers commissioned Harris Interactive (New York) to distribute the survey and incorporate sampling weights based on age, sex, race/ethnicity, education, income, region, marital status, household size and number of children under 18 years of age, so results would be representative of parents of 4-to-8-year-old children in the U.S. Invitations for the Harris Poll Online panel were emailed to a stratified random sample, and respondents were invited to participate in the study with a password-protected email invitation. For parents/caregivers with multiple 4-to-8-year-old children, participants were asked to answer the survey questions about their child with the most recent birthday. Study procedures were reviewed and approved as exempt by the University at Buffalo Institutional Review Board.

### 2.3 Measures

#### 2.3.1 Participant demographics and context

Parents reported on their and their families’ demographics including their age, gender, height, weight, marital status, highest level of education, household income, employment status, race/ethnicity and whether the household received any government benefits (e.g., SNAP or Medicaid). A brief two-item screen (23) was adapted and administered to identify households at risk for food insecurity. Items assess how often the household ‘worried whether food would run out before we got money to buy more’ and how often ‘the food that we bought just did not last and we did not have money to get more’, with response options of: often, sometimes and never. These questions were modified to ask participants about their experiences during the last two months versus the original screen which asks participants about the last 12 months to capture experiences during the pandemic.

Parents were asked questions about the extent of current COVID-19-related protection measures in their town/city, including if mask wearing was mandated and whether there were restaurant-related restrictions. Additionally, children’s schooling and care location in the last week was also assessed (i.e., in-person elementary school, virtual elementary school, home school, and/or in-or out-of-home non-parental child-care).

#### 2.3.2 Child temperament

The Negative Affectivity subscale of the Child Behavior Questionnaire-Very Short Form (CBQ-VSF) was used to assess children’s temperamental negativity (24). Negative Affectivity includes the temperament dimensions of Sadness, Fear, Anger/Frustration, Discomfort, and negative loadings for Falling Reactivity/Soothability. The CBQ-VSF is a reliable and valid parent-report measure of child temperament and includes statements such as “When angry about something, s/he tends to stay upset for 10 min or longer.” Items were rated on a 7-point scale ranging from 1 (extremely untrue of your child) to 7 (extremely true of your child) (24). Parents were also given a not applicable response option for when the child had not been observed in the situation that was described.

#### 2.3.3 Current food acquisition and eating behaviors

Parents completed questions adapted from measures used in previous research, detailing their use of restaurants during the past 2 months (25). Questions included the frequency that the child consumed food from restaurants in-person, via take-out, and via delivery, with response options including: never, once a month or less, 2–3 times a month, once a week, 2–3 times a week, and 4 or more times a week. This question was asked three times to capture each of the three different restaurant contexts: dine-in, take-out, or delivery. For each context, parents also indicated who typically selected the child’s meal (i.e., child, parent and child together, parent, another adult), the most important (1) to least important (7) reasons for the child’s meal choice (e.g., taste, nutrition, habit), and how safe they felt it was to obtain food from a restaurant (i.e., very unsafe, somewhat unsafe, somewhat safe, very safe). These questions were all asked in the context of the past 2 months.

### 2.4 Statistical analysis

We used descriptive statistics to examine frequencies (categorical variables) or means and standard deviations (continuous variables) for all variables of interest. Distributions were also assessed for normality. All analyses incorporated sampling weights, so that results were representative of U.S. parents with 4-to-8-year-old children. Sampling weights were based on parent age, sex, race and ethnicity, education, income, region, marital status, household size, and number of children under 18 years. Linear regression examined whether child negative affectivity and at-home vs. out-of-home childcare/school predicted the frequency the child consumed restaurant meals and the parent-reported reasons for the child’s meal choice, each of which was analyzed in the context of take-out, delivery, and dining in. Analyses of reasons for the meal choice were restricted to those parents who reported some role in that decision (*n* = 413 for dine-in, *n* = 562 for take-out, *n* = 538 for delivery), since those uninvolved in the decision may not know the reasoning that went into the decision. Analyses of reasons were also narrowed to the top three reasons reported for each dining context. Diagnostic plots were used to assess model assumptions. Generally, these plots were satisfactory, but to be conservative, these models were also repeated as ordinal logistic regressions, and the nature of the results was similar.

Logistic regression was used to examine whether child negative affectivity and at-home vs. out-of-home childcare/school predicted who chose the child’s meal in the context of take-out, delivery, and dining in. The following variables were considered covariates in the aforementioned regression models: income, education, employment, race/ethnicity and the level of COVID-19-related restrictions in the participant’s town. Backwards deletion was used to remove covariates that were not statistically significant predictors in each model.

## 3 Results

### 3.1 Demographics and context

A majority of parents reported that their child was attending school virtually (47%) or being homeschooled (31%), 21% of parents reported that their child was attending school in-person, and 19% reported that they were receiving child-care outside of the home/school. The average score on the negative affectivity subscale was 4.0 (SEM = 0.04) (possible range 1–7). Participant demographics are shown in Table 1.

A majority of parents reported eating home-cooked meals more often than before the pandemic (64%), while 22% reported no change from before the pandemic. Parents reported that during the past 2 months, 27% of children were dining-in at restaurants least once a week, while 37% had restaurant food via take-out and 34% via delivery at least once per week. Over half of parents were involved in deciding what their child ordered to eat from restaurants, by either making the meal decision on their own or together with their child. When ranking reasons for choosing the child’s meal, parents who played a role in the decision (46.7%) reported that taste was the most important reason followed by nutrition. Complete descriptive statistics on these food acquisition and eating behaviors are reported in Table 2.

**Table 2:** Parent-reported restaurant use and ordering for their 4-to-8-year-old-child and perceived restaurant safety (*n* = 1000*).

	How often child ate at/from restaurants (past 2 months)		
	In-person (%)	Take-out (%)	Delivery (%)
Never	28	14	23
≤ 1x/month	24	24	20
2 – 3x/month	21	25	23
1x/week	13	19	15
2 – 3x/week	11	14	15
≥4/week	3	4	4

Who typically decided what to order for child (past 2 months)?			
	In-person (%)	Take-out (%)	Delivery (%)
Mother (Reporting parent)	12	16	18
Father (Reporting parent)	17	16	18
The child	33	28	23
Parent & child together	28	34	34
Another adult	5	4	4
Child and another adult	4	2	3

Parent ranking of the importance of different factors when choosing a restaurant meal for their child over the past 2 months (*n* = 467)^a^^,^^b^			
	In-person	Take-out	Delivery
	Mean (SEM)	Mean (SEM)	Mean (SEM)
Taste – child likes the foods in the meal	3.2 (0.1)	3.2 (0.1)	3.3 (0.1)
Habit – what the child typically orders	3.7 (0.1)	3.6 (0.1)	4.1 (0.1)
Cost – price of the meal	4.6 (0.1)	4.5 (0.1)	4.5 (0.1)
Nutrition – health of the meal	3.5 (0.1)	3.6 (0.1)	3.8 (0.1)
Appeal – the meal looks good	4.2 (0.1)	4.2 (0.1)	4.0 (0.1)
Treat – my child does not get it often	4.3 (0.1)	4.1 (0.1)	4.0 (0.1)
New-trying a new flavor	4.5 (0.1)	4.7 (0.1)	4.5 (0.1)

Parents ranked factors shown above on a 7-point scale, where 1 was the most important reason, and 7 was least important. The means depict the average rank for each reason, with lower means indicating that the reason was more important on average.

a All 1,000 parents responded to restaurant frequency questions (for dining in-person, take-out, and delivery). Parents who responded “never” to these were not asked to respond to the subsequent questions about that mode of restaurant use.

b In addition, only parents who reported playing a role in deciding the child’s meal order (determining it themselves or with the child) were included in this analysis, as those not playing any role in the decision would not be expected to know which factors contributed to the decision.

### 3.2 School/childcare status as a predictor of restaurant-related behaviors

Children who received all of their childcare/schooling at home consumed delivery food from restaurants less frequently than children who were receiving some out-of-home childcare or schooling (*p* < 0.01). Childcare/school status was not a significant predictor of the how often the child had food from restaurants via dine-in or take-out, though the nature of these relationships were consistent with delivery (*p* = 0.06 for both) (Table 3). Childcare/school at home was not predictive of who chooses the child’s meal for dine-in (*p* = 0.50), take-out (*p* = 0.15) or delivery (*p* = 0.15).

**Table 3:** Regression results for the frequency of restaurant use and who chose the child’s meal in childcare/school status analysis.

	Dine-in t-statistic (*p*-value)	Take-out t-statistic (*p*-value)	Delivery t-statistic (*p*-value)
Frequency R^2^ 0.06–0.11			
Childcare/school status	−1.90^#^	−1.91^#^	−2.75^**^
Income	3.75^****^	---	2.51^*^
Education	---	5.58^****^	2.23^*^
Employment	3.82^***^	----	---
COVID-19 restrictions	----	---	−2.57^**^
Non-Hispanic Asian	----	----	−2.22^*^
Non-Hispanic Black		----	----
Hispanic	----	----	----
Other race/ethnicity	----	----	----
Who chose the child’s meal			
Childcare/school status	−0.67	−1.43	−1.45
Income	---	---	---
Education	3.32^***^	3.13^**^	2.33^**^
Employment	----	----	----
COVID-19 restrictions	−2.60^***^	−2.87^**^	----
Non-Hispanic Asian	----	---	----
Non-Hispanic Black	----	3.57^***^	----
Hispanic	----	---	----
Other Race/ethnicity	----	---	----

^#^*p* < 0.10, ^*^p < 0.05, ^**^p < 0.01, ^***^p < 0.001, ^****^p < 0.0001. ---- indicates that these covariates were non-significant predictors and thus dropped from the final models. Ranges in R^2^ values represent the R^2^ values for the models examining each of the three different restaurant frequency outcomes: dine-in, take-out, delivery. R^2^ values are not presented for the outcome of who chose the child’s meal given that R^2^ values are not available in the context of logistic regression.

Parents who reported that their child was receiving all of their care and/or schooling at home rated nutrition as more important when rank ordering reasons for selecting the child’s restaurant meal for dine-in (*p* < 0.001) and delivery (*p* < 0.0001) compared to those with children attending school or childcare outside of the home (Table 4).

**Table 4:** Regression results for the parent-reported reasons for selecting a child’s meal in child care/school location analysis.

	Dine-in t-statistic	Take-out t-statistic	Delivery t-statistic
Reason: Taste R^2^ 0.03–0.07			
At home Childcare/school	1.07	1.82^#^	−1.01
Income	----	----	----
Education	3.60^***^	----	----
Employment	----	----	2.99^**^
COVID-19 restrictions	----	−2.86^**^	----
Non-Hispanic Asian	----	----	----
Non-Hispanic Black	2.75^**^	----	----
Hispanic	----	----	----
Other race/ethnicity	----	----	----
Reason: Nutrition R^2^ 0.03–0.05			
At home Childcare/School	−2.30*	−1.33	−2.58**
Income	−2.43*	−4.64***	−2.72**
Education	----	----	----
Employment	----	----	----
COVID-19 Restrictions	----	----	----
Non-Hispanic Asian	----	----	----
Non-Hispanic Black	----	----	----
Hispanic	----	----	----
Other Race/Ethnicity	----	----	----
Reason: Habit R^2^ 0.02–0.04			
At home Childcare/School	0.27	0.52	
Income	----	----	
Education	----	----	
Employment	----	----	
COVID-19 restrictions	−2.34^*^	----	
Non-Hispanic Asian	----	----	
Non-Hispanic Black	----	2.85^*^	
Hispanic	----	----	
Other race/ethnicity	−2.13^*^	----	

^#^*p* < 0.10, ^*^p < 0.05, ^**^p < 0.01, ^***^p < 0.001, ^****^p < 0.0001. ---- indicates that these covariates were non-significant predictors and thus dropped from the final models. The range in R^2^ values shown for each reason represents R^2^ values for the models examining each of the three different restaurant frequency outcomes: dine-in, take-out, delivery. The delivery section for “Reason: Habit” is gray because the top 3 reasons parents endorsed for choice of their child’s delivery meal were taste, nutrition, and treat (not habit). Childcare/school status was a non-significant predictor of the reason that is not included here (treat).

### 3.3 Child temperament as a predictor of restaurant-related behaviors

Parents whose children were higher on negative affectivity reported more frequent child use of restaurants in-person (*p* < 0.05) and for delivery (*p* < 0.05) than parents with children lower on negative affectivity (Table 5). Child negativity did not significantly predict the frequency of getting take-out meals from restaurants or who selected the child’s meal for dine-in, take-out or delivery.

**Table 5:** Regression results for the frequency of restaurant use and who chose the child’s meal in child temperament analysis.

	Dine-in t-statistic (*p*-value)	Take-out-statistic (*p*-value)	Deliveryt-statistic (*p*-value)
Frequency R^2^ 0.05–0.11			
Child temperament	−2.06^*^	1.40	2.39^*^
Income	3.58^***^	---	2.48^*^
Education	---	5.49^****^	---
Employment	3.76^***^	----	---
COVID-19 restrictions	----	---	−2.35^*^
Non-Hispanic Asian	----	----	−2.28^*^
Non-Hispanic Black		----	----
Hispanic	----	----	----
Other race/ethnicity	----	----	----
Who chose the child’s meal			
Child temperament	1.26	0.66	0.64
Income	---	---	---
Education	3.34^***^	3.23^**^	2.40^*^
Employment	----	----	----
COVID-19 restrictions	---	−2.76^**^	----
Non-Hispanic Asian	----	---	----
Non-Hispanic Black	----	3.63^***^	----
Hispanic	----	---	----
Other race/ethnicity	----	---	----

*^#^p* < 0.10, ^*^p < 0.05, ^**^p < 0.01, ^***^p < 0.001, ^****^p < 0.0001. ---- indicates that these covariates were non-significant predictors and thus dropped from the final models. Ranges in R^2^ values represent the R^2^ values for the models examining each of the three different restaurant frequency outcomes: dine-in, take-out, delivery. R^2^ values are not presented for the outcome of who chose the child’s meal given that R^2^ values are not available in the context of logistic regression.

When rank ordering reasons for selecting the child’s restaurant meal, parents whose children were higher on negative affectivity rated taste as a more important reason for child meal selection when ordering restaurant food for delivery (*p* < 0.01), compared to those with children lower on negativity affectivity (Table 6). Full results for school/childcare status and temperament as predictors of the child’s frequency of restaurant use and reasons for meal selection are in Tables 3–6.

**Table 6:** Regression results for the parent-reported reasons for selecting a child’s meal in child temperament analysis.

	Dine-in t-statistic (*p*-value)	Take-out t-statistic (*p*-value)	Delivery t-statistic (*p*-value)
Reason: Taste R^2^ 0.03–0.07			
Child temperament	1.24	1.92^#^	2.49^**^
Income	----	----	----
Education	3.47^***^	----	----
Employment	----	----	3.11^**^
COVID-19 restrictions	----	−2.41^*^	----
Non-Hispanic Asian	----	----	----
Non-Hispanic Black	2.86^**^	----	----
Hispanic	----	----	----
Other race/ethnicity	----	----	----
Reason: Nutrition R^2^ 0.01–0.05			
Child temperament	0.40	−0.99	−2.35^*^
Income	−2.13^*^	−4.48^***^	−2.35^**^
Education	----	----	----
employment	----	----	----
COVID-19 Restrictions	----	----	----
Non-Hispanic Asian	----	----	----
Non-Hispanic Black	----	----	----
Hispanic	----	----	----
Other race/ethnicity	----	----	----
Reason: Habit R^2^ 0.02–0.04			
Child temperament	−0.36	1.04	
Income	----	----	
Education	----	----	
Employment	----	----	
COVID-19 restrictions	−2.33^*^	----	
Non-Hispanic Asian	----	----	
Non-Hispanic Black	----	3.06^**^	
Hispanic	----	----	
Other race/ethnicity	−2.18^*^	----	

Note: ^#^*p* < 0.10, ^*^p < 0.05, ^**^p < 0.01, ^***^p < 0.001, ^****^p < 0.0001. ---- indicates that these covariates were non-significant predictors and thus dropped from the final models. Ranges in R^2^ values represent the R^2^ values for the models examining each of the three different restaurant frequency outcomes: dine-in, take-out, delivery. The delivery section for “Reason: Habit” is gray because the top 3 reasons parents endorsed for choice of their child’s delivery meal were taste, nutrition, and treat (not habit). Child temperament was a non-significant predictor of the reason that is not included here (treat).

### 3.4 Sociodemographics as a predictor of restaurant-related behaviors

Sociodemographic covariates were significant in many of the models, as shown in Tables 3–6, and generally showed that parents with higher income reported more frequent use of restaurants for dine-in and delivery and employed parents reported more frequent use of restaurants for dine-in only. Participants living in an area with more COVID-19-related restrictions reported more frequent use of restaurants for delivery, while non-Hispanic Asian participants reported less frequent use of restaurants for delivery. Sociodemographic covariates were significant when looking at who chose the child’s meal, with parents reporting higher education being more likely to play a role in the child’s meal choice for take-out and delivery.

## 4 Discussion

The present study aimed to better understand the extent to which school/care context and child temperament predicted variability in restaurant-related behaviors during COVID-19. While sociodemographics generally predicted more variability in restaurant-related behaviors than child temperament or childcare/school status, the latter factors were linked with some restaurant behaviors. Both child temperament and childcare/schooling location predicted frequency of restaurant use. Children higher on negative affectivity used restaurants in-person and for delivery more frequently than children with lower negative affectivity, and children receiving childcare/schooling at home used delivery services less frequently than those receiving out-of-home care or schooling. Parents with children higher on negativity rated taste as a more important reason when ordering a delivery meal, which is consistent with previous research findings (25). Overall, these findings suggest that during the early stages of the COVID-19 pandemic, these individual and family factors may have impacted the frequency of restaurant patronage across different modes of restaurant use, as well as the process of selecting children’s restaurant meals. Given prior research suggesting that the quality of children’s dietary intake declines with more consumption from restaurants, as well as evidence of increases in childhood obesity after the start of the pandemic (26), it is important to continue to build our understanding of factors predicting variability in children’s restaurant-related behaviors and to consider next steps for further understanding the potential health implications of these links.

In the present analysis, child negativity and child care/school status predicted the child’s frequency of restaurant use, with higher negativity linked to more frequent dining in-person at restaurants and via delivery and at-home childcare/schooling linked to less delivery from restaurants. Prior research has shown that parents of children higher on temperamental negativity were more likely to use instrumental and emotional feeding methods to soothe or distract the child (22). In the context of COVID-19, recent findings have shown that COVID-19-related life changes were positively associated with mothers rewarding their child’s behavior with food (27). It is possible that, in the face of challenging parent–child interactions, in-person dining as well as delivery may be avenues toward ordering favorite foods to reward the child, soothe the child or get them to engage in desired behaviors. Additionally, due to the possible frustrations and boredom of being at home, families may be more likely to dine-in or pick up food from restaurants. This may be especially true for families with children receiving childcare/schooling at home. However, food acquisition from restaurants using take-out and delivery is understudied both during COVID-19 and in general. Since the start of the pandemic there has been an increase in the use of takeout and delivery from restaurants with 53% of adults saying takeout and delivery is now essential, and 68% noting that they are more likely to use takeout or delivery than before the pandemic (28, 29). The Deloitte restaurants trends report, which surveyed restaurant customers in 2019, 2020, 2021 and 2023, found that the use of takeout and delivery services may be here to stay with 69% of customers in 2023 reporting that they got take-out or delivery at the same rate or more frequently when compared to before the pandemic (30, 31). In addition, the 2019 report found that only 18% of respondents noted that they order takeout/delivery at least once a week while the 2021 report found that 61% of respondents noted delivery/takeout use at least once a week (30, 31). This trend in takeout/delivery use highlights the need for further research into these different modes of restaurant use and implications for future interventions in restaurant contexts.

Children spending more time at home could also impact food insecurity and food acquisition as parents are potentially providing more meals for their children at home. For food insecure families, there is evidence of parents encouraging children to share and eat food with others, as well as child reports of eating less desirable foods because there were no other options (32). The present survey data show that the majority of children were attending school virtually or being homeschooled at the time of data collection, and parents also reported that they were working from home more and taking on a larger role in the care and instruction of their children (9). In the present analysis, parents who reported that their child was receiving care or attending school at home rated nutrition as a more important factor in selecting a restaurant meal, compared to those with children attending school or childcare outside of the home. This may suggest that with COVID-19 restrictions and parents spending more time at home, restaurants were being used to fill the role of regular family meals instead of being used for convenience.

Higher child negativity was also predictive of taste being ranked as a more important reason for selection of the child’s delivery meal, in comparison to families with children lower on negativity. This may support prior research findings that negative affectivity predicts child eating styles such as “picky eating” where children limit their exposure to new foods and trying new tastes (22). This also supports research that shows parents of children who are high in negativity are more likely to feed their child sweet foods and caloric drinks, similar to the foods that tend to characterize children’s menus at restaurants (14, 22). Understanding the interplay between temperament and different aspects of children’s food selection and eating behavior is important given prior research suggesting that aspects of temperament like negative affectivity may be linked to children’s obesity risk (33). Overall, taste has been a key reason for children’s restaurant meal choices outside the context of COVID-19, and future research can examine whether factors motivating meal choices in restaurants differ by child temperament beyond the acute phase of the COVID-19 pandemic (25).

When examining the results from these analyses, the models’ R^2^ values varied, but generally the amount of variance explained by these models was modest. Future research can continue to examine the extent to which these variables are relevant predictors of restaurant-related and other eating behaviors during and beyond the COVID-19 era. Generally, sociodemographics predicted more variability in restaurant-related behaviors than child temperament or childcare/school status (9). It was found that more highly educated and employed parents, as well as parents who reported living in an area with more COVID-19-related restrictions, were all predictive of taste being a more important reason for selecting their child’s meal. Higher income was predictive of ranking nutrition as an important reason for choosing the child’s meal. Families of high socioeconomic status had access to more resources to navigate changes brought about by COVID-19 restrictions, perhaps allowing them to prioritize some of the same reasons for meal choices that have been observed in restaurant research prior to COVID-19 (34). Therefore, families of higher socioeconomic status may continue to view restaurant meals as a “treat” and order more tasty meals or have the ability to order more nutritious meals for their children compared to families of lower socioeconomic status. Overall, variability in these restaurant-related behaviors by sociodemographics highlights the need for further research to inform interventions for those who may be at greatest risk of continued impacts of the COVID-19 pandemic.

Limitations of the present study include the use of a self-report survey measure which may have social desirability bias. Minor modifications were made to some existing survey items as well, for example changing the time frame to the past 2 months, to fit with the aims of the present study. The present study examined individual differences of restaurant-related behaviors but did not collect information about the meals ordered at restaurants. Therefore, health differences are not entirely known, and future studies can work to address this. A strength of this study was the use of sampling weights to create a nationally representative sample of U.S. families with 4-to-8-year-old children. This study was also conducted in October 2020, when many of the initial COVID-19 protection measures and restrictions were relaxed or lifted. Therefore, these findings may highlight longer-term impacts of the COVID-19 pandemic on children’s eating and health as well as individual and family factors which were shown to impact children’s restaurant use and meal selection during COVID-19. The use of restaurant take-out and delivery is understudied both during COVID-19 and in general. These findings suggest the need for additional research, examining the frequency and health implications of these different modes of restaurant use during the COVID-19 era and beyond, and considering the potential for health-related interventions in these contexts.

The impacts of the COVID-19 pandemic on families’ routines and health behaviors may continue beyond the COVID-19 era and may vary by child and family factors. Our prior research demonstrated some variability in restaurant related behaviors during COVID-19, with little known about how parent and child factors predict variability in families’ restaurant use (9). The current analysis found that both child negative affectivity and child care/school status predicted the child’s frequency of restaurant use, with higher negativity linked to more frequent dining in-person at restaurants and via delivery and at-home childcare/schooling linked to less delivery from restaurants. Continued research in this area can help us understand differential experiences during COVID-19, the extent to which these persist, and potential corresponding health implications and intervention opportunities. In addition, increased use of restaurants via take-out and delivery may be here to stay, suggesting the need for additional research on these modes of restaurant use. Additional research in these areas can inform intervention and policy approaches that are in alignment with current contexts and different families’ needs.

## Data availability statement

The original contributions presented in the study are included in the article/supplementary material, further inquiries can be directed to the corresponding author.

## Ethics statement

The study was approved by the University at Buffalo Institutional Review Board (STUDY00004723, 8 September 2020). The studies were conducted in accordance with the local legislation and institutional requirements. The participants provided their written informed consent to participate in this study.

## Author contributions

JG: Writing – original draft, Writing – review & editing. MF: Writing – review & editing. ST: Writing – review & editing. LE: Writing – review & editing. LL: Writing – review & editing. SA-F: Writing – original draft, Writing – review & editing.
